# Are orthodontic randomised controlled trials justified with a citation of an appropriate systematic review?

**DOI:** 10.1186/s40510-021-00395-z

**Published:** 2021-12-17

**Authors:** Kishan Patel, Martyn T. Cobourne, Nikolaos Pandis, Jadbinder Seehra

**Affiliations:** 1 Department of Orthodontics, Faculty of Dentistry, Oral and Craniofacial Sciences, King’s College London, Kings College Hospital NHS Foundation Trust, Denmark Hill, London, SE5 9RS UK; 2 Department of Orthodontics and Dentofacial Orthopedics, Dental School/Medical Faculty, University of Bern, Bern, Switzerland; 3 Centre for Craniofacial Development and Regeneration, Faculty of Dentistry, Oral and Craniofacial Sciences, King’s College London, Guy’s Hospital, Guy’s and St Thomas NHS Foundation Trust, London, SE1 9RT UK

**Keywords:** Orthodontics, Systematic reviews, Interventional studies, Research waste, SPIRIT, CONSORT

## Abstract

**Background:**

A systematic review of the evidence should be undertaken to support the justification for undertaking a clinical trial. The aim of this study was to examine whether reports of orthodontic Randomised Clinical Trials (RCTs) cite prior systematic reviews (SR) to explain the rationale or justification of the trial. Study characteristics that predicated the citation of SR in the RCT report were also explored.

**Material and methods:**

Orthodontic RCTs published between 1st January 2010 to 31st December 2020 in seven orthodontic journals were identified. All titles and abstracts were screened independently by two authors. Descriptive statistics and associations were assessed for the study characteristics. Logistic regression was used to identify predicators of SR inclusion in the trial report.

**Results:**

301 RCTs fulfilling the eligibility criteria were assessed. 220 SRs were available of which 74.5% (*N* = 164) were cited, and 24.5% (*N* = 56) were not included but were available in the literature within 12 months of trial commencement. When a SR was not included in the introduction or no SR was available within 12 months of trial commencement, interventional studies were commonly cited. The continent of the corresponding author predicated the possibility of inclusion of a SR in the introduction (OR 0.36; 95% CI 0.18–0.71; *p* = 0.003).

**Conclusions:**

A quarter of orthodontic RCTs (24.5%) included in this study did not cite a SR in the introduction section to justify the rationale of the trial when a relevant SR was available. To reduce research waste and optimal usage of resources, researchers should identify or conduct a systematic review of the evidence to support the rationale and justification of the trial.

## Introduction

With a wealth of trials being published in orthodontics, it is incumbent on researchers to ensure transparent reporting of interventional studies. Research waste is a known phenomenon and is a direct product of poorly conducted and reported studies as well as unnecessary duplication [1]. Specific concerns include biased or incomplete reporting and failure to adequately address questions of relevant clinical interest [2]. To aid transparent reporting, established, evidence-based checklists and guidelines have been published to aid authors. Examples of such checklists include the Consolidated Standards of Reporting Trials (CONSORT) [3] and the Standard Protocol Items: Recommendations for Interventional Trials (SPIRIT) statements [4]. There is an ethical and fiduciary responsibility on researchers to contextualise their study within the known realms of the established literature. This is highlighted in both the CONSORT and SPIRIT statements, indicating the citation of an appropriate systematic review (SR) as justification for the undertaking of the trial [3, 4]. The latter specifically states placing: “the trial in the context of the available evidence, it is strongly recommended that an up-to-date SR of relevant studies be summarised and cited in the protocol”. Furthermore, the United Kingdom Health Research Authority has seconded these statements indicating that clinical trial design “should be underpinned by a SR of the existing evidence”, with the primary research question based also upon this [5].

Meta-epidemiological studies aiming to ascertain the proportion of interventional trials having cited an appropriate SR have indicated that many trials still do not cite a relevant SR in their introduction as a justification for its undertaking [6–8]. Neither has this improved with successive reporting, highlighting the need for trial contextualisation [7, 9, 10]. Whilst these studies considered medical journals, a recent study conducted attempted to quantify proper citation of SRs in dental specialty journals [11]. The study indicated that only 62.5% of published and available randomised controlled trials (RCTs) had appropriate citation of a published and relevant SR. Significant factors predicting the citation of an appropriate SR included an increase in the journal impact factor in which the study was published and location of corresponding author, with those located in Europe having more appropriate SR citations. Whilst this study included two orthodontic journals, there is no study exclusively investigating these parameters in the established orthodontic literature. Therefore, the aim of this meta-epidemiological study was to assess the extent to which reports of orthodontic RCTs cite prior SRs to explain the rationale or justification of the trial. Study characteristics that predicted the citation of SR in the RCT report were explored.

## Materials and methods

### Eligibility criteria

Orthodontic RCTs published between a 10-year period (1st January 2010 to 31st December 2020) were sourced from the following seven orthodontic journals: American Journal of Orthodontics and Dentofacial Orthopaedics (AJODO), European Journal of Orthodontics (EJO), Journal of Orthodontics (JO), Angle Orthodontist (ANGLE), Orthodontics and Craniofacial Research (OCR), Journal of Orofacial Orthopedics (JOO) and Australasian Orthodontic Journal (AOJ).

The phrase ‘‘randomised controlled trial’’ was screened in the title, abstract and methodology of the article. In accordance with the Cochrane criteria for the selection of RCTs, the following inclusion criteria was used: human participants, interventions related to healthcare, experimental studies, presence of a control or comparative group, randomisation of participants to control and treatment groups, other trials with terminology in the title or abstract such as ‘‘prospective’’, ‘‘comparative’’, ‘‘efficacy’’ or where an indication was given that a comparison of treatment groups was undertaken prospectively were analysed to establish whether randomisation was implemented. Studies published in English were only included. Case reports, review articles, editorials, systematic reviews and retrospective studies were excluded.

### Selection of studies

Both journal websites and a single electronic database (Medline via PubMed: https://pubmed.ncbi.nlm.nih.gov/) were searched by one author (KP) to identify eligible trials. All titles and abstracts were screened independently by 2 authors (KP and JS). Full-text articles of abstracts fulfilling the inclusion criteria were retrieved and further analysed for eligibility independently by 2 authors (KP and JS). Any disagreements in the final articles were resolved by discussion among the authors.

### Data extraction

A pilot assessment of ten RCTs was undertaken between two authors (KP and JS) to ensure consistency in data extraction variables. All study characteristics were extracted by a single author (KP) and entered into a pre-piloted Microsoft Excel® (Microsoft, Redmond, WA) data collection sheet. A second author (JS) independently cross-checked the collected data. Any discrepancies were resolved by discussion.

At the level of each RCT, the following study characteristics were extracted: year of publication, number of authors, continent of corresponding author (Europe, Americas, Asia and other), journal impact factor (www.clarivate.com/webofsciencegroup/solutions/journal-citation-reports/), journal title, ethical approval (no approval, exempt from approval or ethical approval obtained), involvement of statistician (not reported or reported; inferred from author affiliations and materials and methods section), study registration (no or yes), significance of results (either yes or no based on primary outcome. In the absence of no clear primary outcome, the first outcome was analysed: significant or non-significant), conflict of interest (conflicts exist and declared, no conflicts to declare or not clearly declared) and funding (industry funded and declared, no industry sponsorship/funding to declare or not clearly declared). As recommended by both the CONSORT [3] and SPIRIT [4] checklists, the introduction section of each trial was inspected for the citation of a SR used to justify the rationale of the trial and relevant to the primary trial outcome (yes or no). If no SR was cited, then the literature was searched to identify if a SR was available 12 months prior to the date of trial commencement (yes or no). Also, in the absence of a SR the type of study cited to justify the rationale of the trial was recorded (in-vitro, interventional, observational or none).

### Statistics

Descriptive statistics and associations were calculated for the inclusion of a SR in the introduction, SR not included but available in literature within 12 months of trial commencement and study characteristics. Logistic regression was used to assess associations between SR inclusion in the introduction and the study characteristics. Odd ratios, corresponding 95% CIs and p-values were calculated. Significant predictors identified during the univariate analysis were entered individually in the multivariable modell. In addition, the Boruta feature selection algorithm in R [12] was used as a an alternative method for variable selection using 100 iterations. A two-tailed *p* value of 0.05 was considered statistically significant. All analyses were performed using Stata 16.1 (Stata Corp, TX, USA) and R Software version 4.0.3 (R Foundation for Statistical Computing, Vienna, Austria).

## Results

**Fig. 1:**
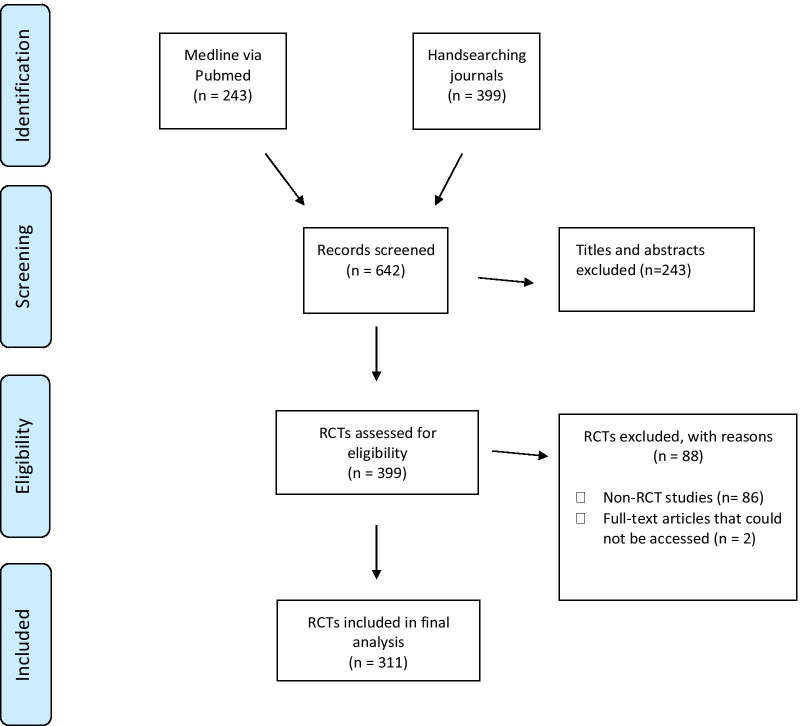
RCT identification flow diagram

A total of 301 RCTs were analysed in this study (Fig. 1). A total of 220 SRs were available of which 74.5% (*N* = 164) were included in the introduction section, and 24.5% (*N* = 56) were not included but were available in the literature within 12 months of trial commencement (Table 1). When a SR was not included in the introduction or no SR was available within 12 months of trial commencement, interventional studies were commonly cited (74.1%) (Table 1).

**Table 1:** The inclusion of a systematic review: (1) included in introduction, (2) available in literature within 12 months of trial commencement but not included and (3) Type of studies included when no systematic review was included in the introduction

		N (%)
(1) SR included in introduction	Yes	164 (74.5%)
(2) SR available in literature within 12 months of trial commencement but not included	Yes	56 (24.5%)
	Total	220 (100%)
(3) Type of study included if no SR available	In-vitro	8 (9.9)
	Interventional	60 (74.1)
	Observational	4 (4.9)
	No study cited	9 (11.1)
	Total	81 (100%)

The characteristics of trials which included SR in the introduction or did not include a SR when there was a SR within 12 months of participant recruitment were compared (Table 2). Within this sub-group, SRs were more likely to be included if the RCT was published in 2020 (75.6%), published in the EJO (76.1%), and had a corresponding author based in Europe (80.0%). When SRs were included, the median number of authors and impact factor were 4.5 and 1.96, respectively. An association between continent of corresponding author (*p* = 0.01) and SR inclusion was detected (Table 2).

**Table 2:** The characteristics of trials which included SR in the introduction or not included when there was a SR available in the literature within 12 months of trial commencement (*N* = 220)

Variable	SR not included but available in literature *N* (%) (*N* = 56)	SR included *N* (%) (*N* = 164)	Pearson Chi^2^ or Fisher’s exact test (*p* < 0.05)
Year of publication			
2010	2 (25.0)	6 (75.0)	0.75
2011	3 (37.5)	5 (62.5)	
2012	4 (40.0)	6 (60.0)	
2013	2 (20.0)	8 (80.0)	
2014	4 (33.3)	8 (66.7)	
2015	5 (27.8)	13 (72.8)	
2016	8 (40.0)	12 (60.0)	
2017	5 (17.9)	23 (82.1)	
2018	5 (16.7)	25 (83.3)	
2019	7 (22.6)	24 (77.4)	
2020	11 (24.4)	34 (75.6)	
Journal type			
AJODO	12 (28.6)	30 (71.4)	0.14
ANGLE	12 (21.1)	45 (78.9)	
EJO	17 (23.9)	54 (76.1)	
OCR	4 (25.0)	12 (75.0)	
AOJ	3 (100.0)	0 (0.0)	
JOO	2 (28.6)	5 (71.4)	
JO	6 (25.0)	18 (75.0)	
Continent of corresponding author			
Europe	22 (20.0)	88 (80.0)	0.006
Americas	5 (14.7)	29 (85.3)	
Asia or other	29 (38.2)	47 (61.8)	
Ethical approval			
No approval or exempt from approval	5 (35.7)	9 (64.3)	0.36
Ethical approval obtained	51 (24.8)	155 (75.2)	
Involvement of statistician			
Not reported	37 (24.5)	114 (75.5)	0.63
Reported	19 (27.5)	50 (72.5)	
Trial registration			
No	37 (28.0)	95 (72.0)	0.28
Yes	19 (21.6)	69 (78.4)	
Significance of results			
Not significant	28 (25.5)	82 (74.5)	1.00
Significant	28 (25.5)	82 (74.5)	
Conflict of interest			
Conflicts exist and declared	33 (26.4)	92 (73.6)	0.85
No conflicts to declare	22 (24.7)	67 (75.3)	
Not clearly declared	1 (16.7)	5 (83.3)	
Funding			
Industry funded and declared	19 (20.7)	73 (79.3)	0.23
No industry sponsorship/funding to declare	30 (31.3)	66 (68.8)	
Not clearly declared	7 (22.6)	24 (77.4)	
Number of authors			
Median (IQR)	5 (3)	4.5 (3)	
Impact factor			
Median (IQR)	1.96 (0.41)	1.96 (0.47)	

**Fig. 2:**
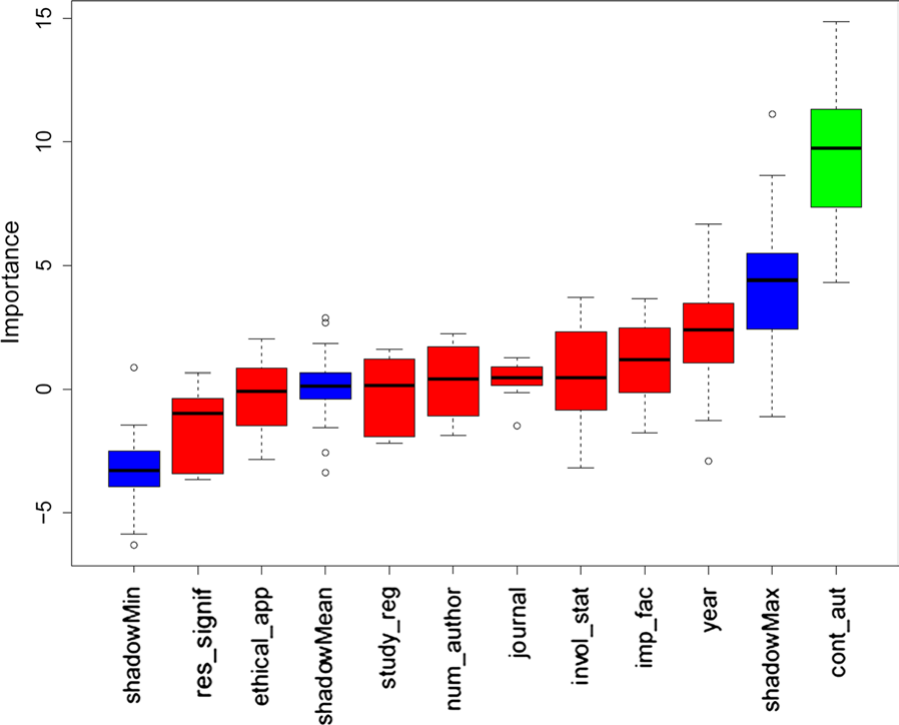
Boruta result plot for the data. Blue boxplots correspond to minimal, average and maximum Z score (importance) of a shadow attribute. Red and green boxplots represent Z scores of, respectively, rejected and confirmed attributes

**Fig. 3:**
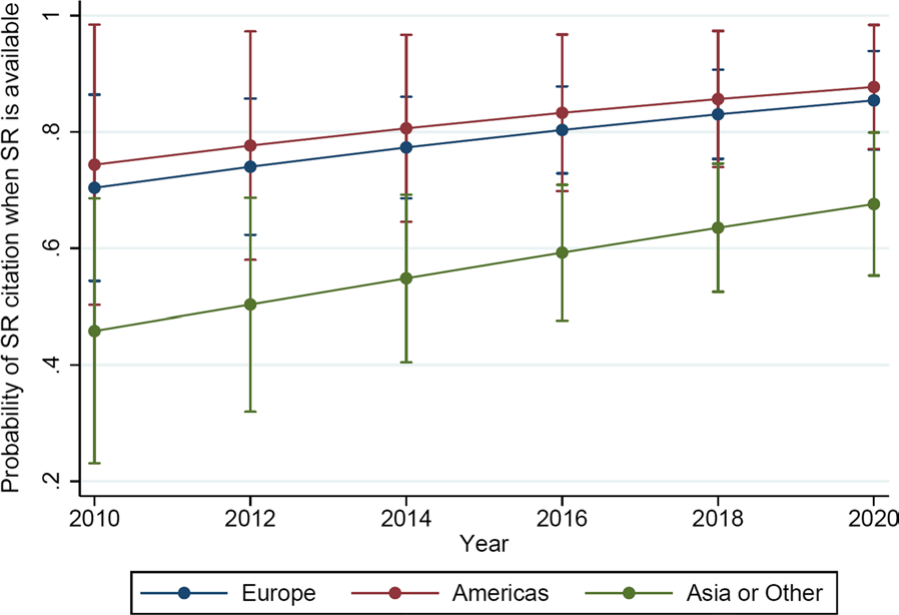
Probability of SR inclusion per continent of corresponding author and year of publication

The Boruta algorithm confirmed continent as an important feature and all the other attributes as not important (Fig. 2). In the final model, continent and year of publication (year as an a priori confounder) were included. In the multivariable analysis, the continent of the corresponding author predicated the possibility of inclusion of a SR in the introduction with authors based in Asia or other having lower odds than those based in Europe (OR: 0.36; 95% CI 0.18–0.71; *p* = 0.003) (Fig. 3; Table 3).

**Table 3:** Univariable and multivariable logistic regression derived odds ratios (OR) and 95% confidence intervals (CI) for the effect of year of publication, journal, continent of corresponding author, ethical approval, involvement of statistician, trial registration, significance of results, conflict of interest, funding, impact factor and number of authors on the likelihood of SR inclusion

Predictor variables	Category	Univariable analysis		Multivariable analysis	
		OR (95%CI)	p-value	OR (95%CI)	p-value
Journal	AJODO	Reference			
	ANGLE	1.50 (0.59, 3.78)	0.390		
	EJO	1.27 (0.54, 3.01)	0.587		
	OCR	1.20 (0.32, 4.47)	0.786		
	AOJ	Not estimable	–		
	JOO	Dropped (exactly same % with AJODO)	–		
	JO	1.00 (0.17, 5.88)	1.00		
Continent of corresponding author	Europe	Reference			
	Americas	1.45 (0.50, 4.18)	0.491	1.22 (0.41, 3.61)	0.72
	Asia or other	0.41 (0.21, 0.78)	0.007	0.36 (0.18, 0.71)	0.003
Ethical approval	No approval or exempt from approval	Reference			
	Ethical approval obtained	1.69 (0.54, 5.27)	0.367		
Involvement of statistician	Not reported	Reference			
	Reported	1.08 (0.66, 1.76)	0.755		
Trial registration	No	Reference			
	Yes	1.41 (0.75, 2.67)	0.284		
Significance of results	Not significant	Reference			
	Significant	1.00 (0.55, 1.83)	1.000		
Conflict of interest	Conflicts exist and declared	Reference			
	No conflicts to declare	1.09 (0.56, 2.04)	0.782		
	Not clearly declared	1.79 (0.20, 15.92)	0.600		
Funding	Industry funded and declared	Reference			
	No industry sponsorship/funding to declare	0.57 (0.29, 1.11)	0.100		
	Not clearly declared	0.89 (0.33, 2.38)	0.820		
Year of publication	Per unit	1.07 (0.96, 1.18)	0.220	1.09 (0.98, 1.22)	0.11
Impact factor	Per unit	1.52 (0.76, 3.06)	0.237		
Number of authors	Per unit	1.07 (0.93, 1.22)	0.339		

## Discussion

Evidence-based checklists and guidelines aimed at promoting transparency of Randomised controlled trials (RCTs) such as the CONSORT and SPIRIT statements have strongly suggested that a systematic review (SR) is cited within the study’s introduction to justify its undertaking [3, 4]. This study identified that almost three-quarters (74.5%) of orthodontic RCTs published from January 2010 to December 2020 had cited a relevant SR within its introduction, with 24.5% of studies not citing one when one was publicly available. A SR was more likely to be cited if the country of the first author was based in Europe. The continent of primary author was deemed to be the only predictive factor for positive SR citation.

This study showed that a higher proportion of orthodontic RCTs cite relevant SRs when compared to dental specialty journals in its entirety. The latter cohort of RCTs demonstrated that only 62.5% of RCTs have a positive SR citation [11]. Additionally, articles published in the European Journal of Orthodontics, within the field of orthodontics in general and when authors were based in Europe were more likely to cite an appropriate SR [11]. This was concurrent with findings of this study. Extrapolating this outside the field of dentistry, many medical interventional studies are being inappropriately justified with reported prevalence of 46–49.5% of RCTs including the appropriate SR to inform trial implementation [6, 13]. Concerning trial protocols, a not grossly dissimilar 40.6% used an SR to inform their trial design [8]. Assessing justification through time, a series of publications auditing the contextualisation of trial findings within the wider literature found no improvement in compliance with established reporting guidelines [7, 9, 10, 14, 15]. Within the current sample, when a SR was not included within the introduction, the most common type of study cited was an interventional one. This is once again, what was found elsewhere in the established literature [11]. A large meta-epidemiological study spanning all areas of medicine attempted to address justification of trial selection via citation of an appropriate interventional study. The results were once again alarming with less than 25% of trials citing an appropriate preceding trial [16].

The responsibility to reduce potentially wasteful research lies on all the stakeholders involved in its conception, design, implementation and dissemination. One specific recommendation made by Glasziou et al. [1] to minimise research waste and undue, unethical and unfounded duplicity of research was to encourage wide adoption of established trial checklists. It is incumbent on journal editors and researchers alike to ensure stringent adoption of such statements as it leads to evidence-based improvement in quality of reporting and overall justification of interventional trials [17]. Medical journals such as the Lancet have already ensured that prospective authors willing to submit to their journal follow such checklists [10]. Furthermore, orthodontic journals should take lead in enforcing SR citation in informing trial conception. Indeed, some leading medical journals require pre-publication of the trial protocol before they can consider an RCT for publication. Possibly, the publication of the protocol will aid researchers in considering the available evidence such as a SR before undertaking a trial [18, 19]. Other methods to improve contextualisation of existing literature include targeting research and ethics committees and ensuring that appropriate due diligence has been undertaken in the form of SR citation prior to trial approval.

The EU clinical trial regulation has made strides in minimising sub-optimal research with its position statement which is due to come into effect in 2021/2022, arming local research and ethics committees alongside national competent authorities to disregard redundant research proposals. They have suggested that ‘applicants for trial authorisation shall justify a new proposal that addresses and *outstanding clinical uncertainty* in light of the available evidence relevant for the research question and the outcome of interest at issue’. Whilst it does not specifically mention the need for a SR, it does go on to mention that ‘where no systematic review exists, applicants should make their best efforts to identify and synthesise knowledge gained in prior studies’ [20]. As recommended in both the CONSORT and SPIRIT checklists, prior to carrying out a Randomised Clinical Trial, undertaking a systematic review is an important step to identify any pre-existing primary trials and hence support the justification of the trial and avoid research wastage.

The methodology of the present study was based on the recommendations of the SPIRIT guidelines [4] which state that a trial report should cite a relevant and recent SR to justify the rationale of the study. However, this may be associated with a degree of bias as it could be difficult to differentiate between RCTs that cited a SR to inform the trial and RCTs that also cited a SR but did not explain its impact on the trial design. This study has highlighted that almost a quarter of RCTs did not cite a SR when there was a review available in the literature within 12 months of trial commencement. Previous studies have reported the number RCTs citing a relevant SR as a proportion of the total number of RCTs sampled. However, adopting this approach may result in an overestimation of the situation. To avoid potential bias, the inclusion of SRs available within 12 months of the trial commencement has been recommended [8].

Orthodontic RCTs published between 2010 and 2020 were only included in this study. Within this timeframe, three hundred and one RCTs were identified which represent a large enough sample to ascertain if SR are cited in the reports of RCTs. Whilst all attempts were made by the authors to ensure rigorous literature search, some studies may have been missed. This may be compounded by the fact that RCT articles were limited to English language only, hence potentially eligible RCTs may have been excluded leading to potential bias. However, through independent assessment by two authors every attempt was made to identify SRs which correlate with the primary aim of the trial and reduce potential selection bias.

## Conclusion

As per evidence-based checklists such as CONSORT and SPIRIT statements, almost a quarter (24.5%) of RCTs did not cite an appropriate SR within the introduction section as justification for the trial, when one was available. Trials where the corresponding author was based in Europe were the only predictive factor identified for positive SR citation. Further work by all research stakeholders is required within the field of orthodontics to limit research waste, ensuring finite resources are corralled for appropriately justified trials.

## Data Availability

Not applicable.
